# Plasma fibrinogen levels are correlated with postoperative distant metastasis and prognosis in esophageal squamous cell carcinoma

**DOI:** 10.18632/oncotarget.4800

**Published:** 2015-08-04

**Authors:** Danhong Zhang, Xia Zhou, Wuan Bao, Ying Chen, Lei Cheng, Guoqin Qiu, Liming Sheng, Yongling Ji, Xianghui Du

**Affiliations:** ^1^ Department of Radiotherapy, Zhejiang Cancer Hospital, Hangzhou, Zhejiang, China; ^2^ Key Laboratory Diagnosis and Treatment Technology on Thoracic Oncology, Zhejiang, China

**Keywords:** esophageal squamous cell carcinoma, fibrinogen, metastasis, prognosis

## Abstract

This study investigated the correlation of preoperative plasma fibrinogen level with distant metastasis and prognosis in esophageal squamous cell carcinoma (ESCC). A total of 255 patients with ESCC who underwent surgery in Zhejiang cancer hospital (Hangzhou, China), between October 2006 and December 2009, were evaluated in this retrospective study. Population controls were selected from a pool of cancer-free subjects in the same region. Each patient and cancer-free people provided 3-mL pretreatment blood. Plasma fibrinogen level was measured by the Clauss method. The effects of hyperfibrinogenemia on locoregional relapse-free survival (LRFS), distant metastasis-free survival (DMFS), relapse-free survival (RFS), and overall survival (OS) were assessed using Kaplan-Meier analysis. Independent prognostic factors were identified in the multivariate Cox analysis. The proportion of hyperfibrinogenemia was higher in ESCC patients than those in controls (40.4% vs 13.6%). Subjects with hyperfibrinogenemia had a significantly higher risk of ESCC than those with normal plasma fibrinogen level (adjust OR = 4.61; 95% CI = 3.02–7.01, *P* < 0.001) after adjusted for age, sex and smoking status. The Kaplan-Meier curves showed that patients with hyperfibrinogenemia had worse DMFS, RFS and OS (*P* < 0.001). Tumor length, lymph node metastasis and plasma fibrinogen level were independent prognostic factors of ESCC (*P* < 0.05). Increased plasma fibrinogen level was significantly associated with elevated risk of ESCC. Preoperative plasma fibrinogen level was a predictor of distant metastasis and independently associated with prognosis of patients with ESCC.

## INTRODUCTION

Esophageal cancer is one of the common malignant neoplasms, with very poor outcome. Squamous cell carcinoma (ESCC) accounts for 80% of all esophageal cancer in Eastern countries [1]. Despite the great progress of in the management of operable ESCC, the prognosis for patients with distant organ metastasis is still unsatisfactory [2]. 20–30% of patients are newly diagnosed with ESCC will have distant metastasis. Liver, lung, and bones are the common sites of metastasis [3]. Up to now, the American Joint Committee on Cancer (AJCC) staging system is commonly used to predict prognosis for patients with ESCC. However, some patients with similar clinical stage have remarkably different survival prognosis. In this way, heterogeneity of protein expression profiles may play a very important role in the development of ESCC [4]. The ability to predict patients with high risk of distant organ metastasis would help guide adjuvant chemotherapy or radiotherapy treatment during the decision-making process. To date, most of these markers had not been proven to be sufficiently effective [5].

Recently, Considerable attention has been given to the relationship between cancer metastasis and coagulation. Cancer can cause a hypercoagulable state and this complex system seems to contribute to cancer progression. The incidence of coagulation abnormalities exceeds 50% in cancer patients. To a certain extent, cancer behaves like a wound which never heals [6]. Hypercoagulability is a sign of a more aggressive disease. D-dimers [7] and platelets [8] are reorted to to be correlated with tumor stage, regional lymph nodes involvement and prognosis. In order to analyze if hyperfibrinogenemia is associated with host susceptibility to ESCC, we describe a case–control study of 255 ESCC cases and 273 controls in a southeast Chinese population. Furthermore, we aimed to determine the effects of preoperative plasma fibrinogen level on relapse (locoregional relapse, distant metastasis, and overall recurrence) and survival (locoregional relapse-free survival, distant metastasis-free survival, relapse-free survival, and overall survival).

## RESULTS

**Figure 1:**
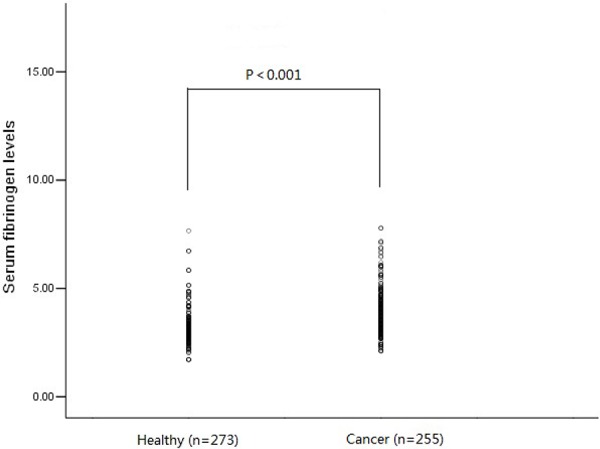
Plasma fibrinogen level in ESCC patients (*n* = 255) was significantly higher than that of healthy controls (*n* = 273) (3.89 ± 1.02 g/L vs 3.21 ± 0.84 g/L, *P* < 0.001)

A total of 255 ESCC patients and 273 healthy controls were enrolled in our study. The median age was 57 (range, 36–81 years) for cancer patients and 58 (range, 37–76) for control subjects. There were no statistically significant differences among cases and controls in terms of age and sex distributions. Of the 255 ESCC patients, 41 were non-smokers and 214 were former or current smokers. These variables had similar proportions in 273 healthy controls. Hence, no statistically significant differences were found compared with those in the cases. Plasma fibrinogen level was examined in all ESCC patients and healthy population. Plasma fibrinogen level in ESCC patients was significantly higher than that of healthy controls (*P* < 0.001, Figure 1). The proportion of hyperfibrinogenemia was higher in ESCC patients than those in controls (40.4% vs 13.6%). Subjects with hyperfibrinogenemia had a significantly higher risk of ESCC than those with normal plasma fibrinogen level (adjust OR = 4.61; 95% CI = 3.02–7.01, *P* < 0.001) after adjusted for age, sex and smoking status.

The baseline characteristics of these ESCC patients are summarized in Table 1. The median of plasma fibrinogen concentration in all patients was 3.89 g/L (range: 2.11–7.80 g/L). Plasma fibrinogen level was significantly associated with gender (*P* = 0.018), tumor location (*P* = 0.012), tumor length (*P* < 0.001), T stage (*P* < 0.001) and N stage (*P* < 0.001), whereas there was no significant association between plasma fibrinogen level and age, smoking history, alcohol history and tumor cell differentiation (*P* < 0.05).

**Table 1:** Plasma fibrinogen level and clinicopathological characteristics in 255 ESCC patients

Variables	Patients (*n*)	Plasma fibrinogen level		*P*
		Normal (<4 g/L)	High (>4 g/L)	
Gender				
Female	23	19 (82.6)	4 (17.4)	**0.018**
Male	232	133 (57.3)	99 (42.7)	
Age (years)				
≥65 Y	31	23 (74.2)	8 (25.8)	0.077
<65 Y	224	129 (57.6)	95 (42.4)	
Smoking				
Never	41	29 (70.7)	12 (29.3)	0.113
Ever	214	123 (57.5)	91 (42.5)	
Alcohol				
Never	72	49 (68.1)	23 (31.9)	0.085
Ever	183	103 (56.3)	80 (43.7)	
Differentiation				
Well	30	20 (66.7)	10 (33.3)	0.664
Moderately	179	104 (58.1)	75 (41.9)	
Poorly	46	28 (60.9)	18 (39.1)	
Tumor location				
Upper	6	3 (50.0)	3 (50.0)	**0.012**
Middle	123	85 (69.1)	38 (30.9)	
Lower	126	64 (50.8)	62 (49.2)	
Tumor length				
≤5 cm	197	135 (68.5)	62 (31.5)	**<0.001**
>5 cm	58	17 (29.3)	41 (70.7)	
T stage				
T1	36	32 (88.9)	4 (11.1)	**<0.001**
T2	49	39 (79.6)	10 (20.4)	
T3	151	75 (49.7)	76 (50.3)	
T4	19	6 (31.6)	13 (68.4)	
N stage				
N0	127	89 (70.1)	38 (29.9)	**0.004**
N1	74	40 (54.1)	34 (45.9)	
N2	38	17 (44.7)	21 (55.3)	
N3	16	6 (37.5)	10 (62.5)	
Locoregional relapse				
Yes	32	21 (65.6)	11 (34.4)	0.458
No	223	131(58.7)	92(41.3)	
Distant metastasis				
Yes	92	37(40.2)	55(59.8)	**<0.001**
No	163	115(70.6)	48(29.4)	
Any relapse				
Yes	121	55(45.5)	66(54.5)	**<0.001**
No	134	97(72.4)	37(27.6)	
Deaths				
Yes	81	34(42.0)	47(58.0)	**<0.001**
No	174	118(67.8)	56(32.2)	

Bold values are statistically significant (*P* < 0.05).

After a median follow-up time of 37 months, 32 patients (12.5%) underwent locoregional relapse, 92 (36.1%) had distant metastasis, 121 (47.5%) had treatment failure and 81 (31.8%) died among 255 ESCC patients. The 5-year LRFS, DMFS, RFS and OS rates were 75.0%, 46.9%, 35.1% and 53.5%, respectively. Distant metastasis was diagnosed in 53.3% (55/103) of patients with hyperfibrinogenemia versus 24.3% (37/152) of patients with normal plasma fibrinogen level (*P* < 0.001). For any relapse, the percentage was 64.1% (66/103) versus 36.2% (55/152) (*P* < 0.001). Mortality was 45.6% (47/103) in patients with hyperfibrinogenemia versus 22.4% (34/152) in patients with normal plasma fibrinogen level (*P* < 0.001) (Table 1). The locoegional relapse rate was not significantly different between patients with hyperfibrinogenemia and patients with normal plasma fibrinogen level.

**Figure 2:**
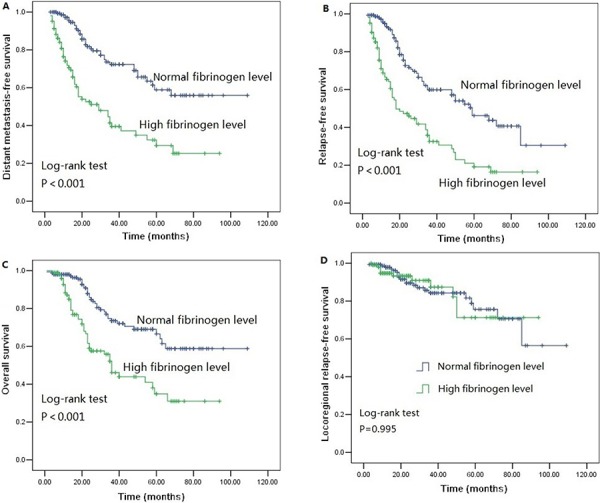
Survival curves of **A.** distant metastasis free survival (DMFS) subdivided by plasma fibrinogen level in ESCC patients (*n* = 255), **B.** Relapse-free survival (RFS) subdivided by plasma fibrinogen level in ESCC patients (*n* = 255), **C.** Overall survival (OS) subdivided by plasma fibrinogen level in ESCC patients (*n* = 255), **D.** Locoregional relapse-free survival (LRFS) subdivided by plasma fibrinogen level in ESCC patients (*n* = 255).

**Figure 3:**
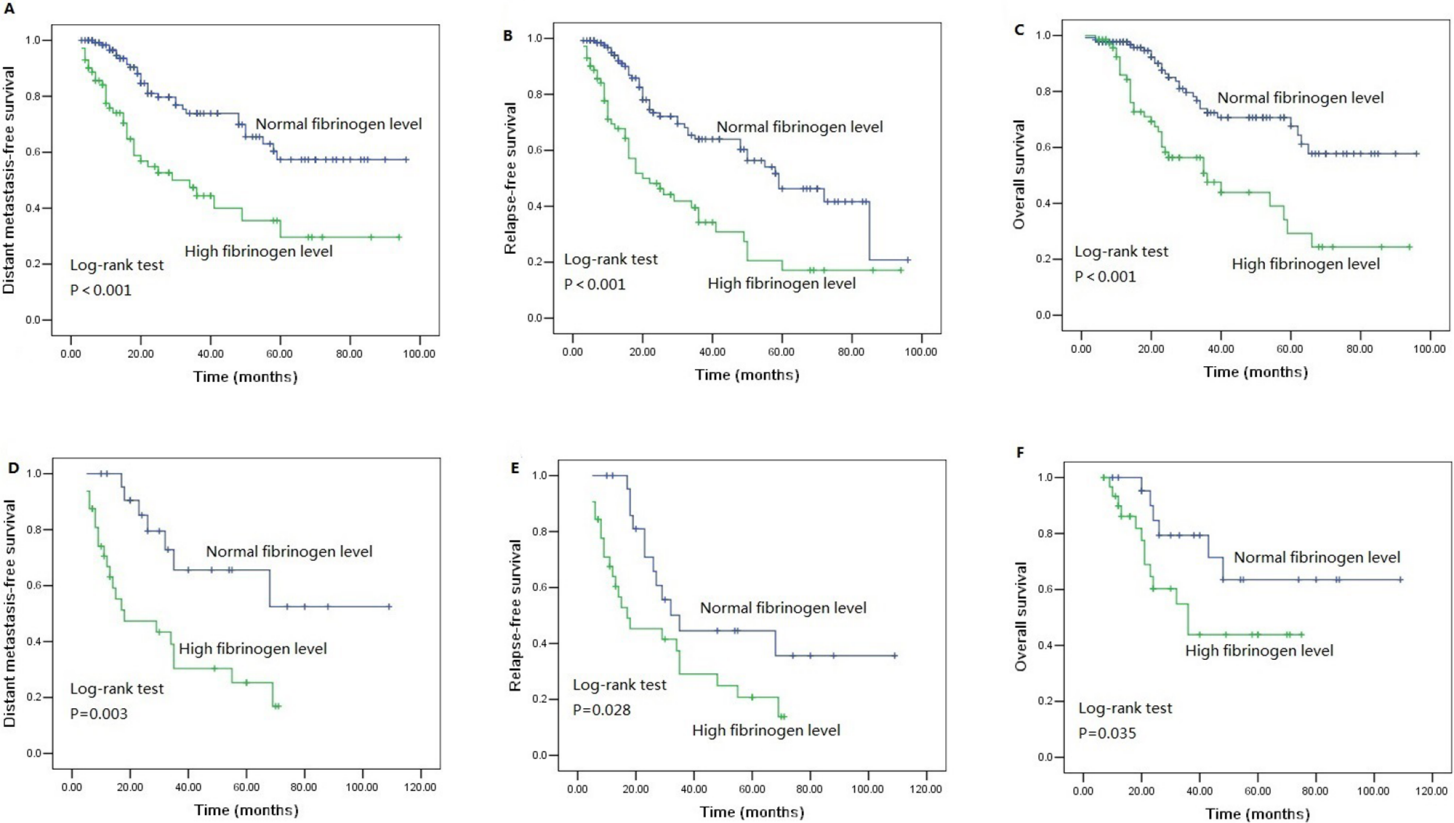
Survival curves of **A.** DMFS **B.** RFS **C.** OS subdivided by plasma fibrinogen level in ESCC patients without adjuvant postoperative chemotherapy (*n* = 200), **D.** DMFS **E.** RFS **F.** OS subdivided by plasma fibrinogen level in ESCC patients with adjuvant postoperative chemotherapy (*n* = 55).

**Figure 4:**
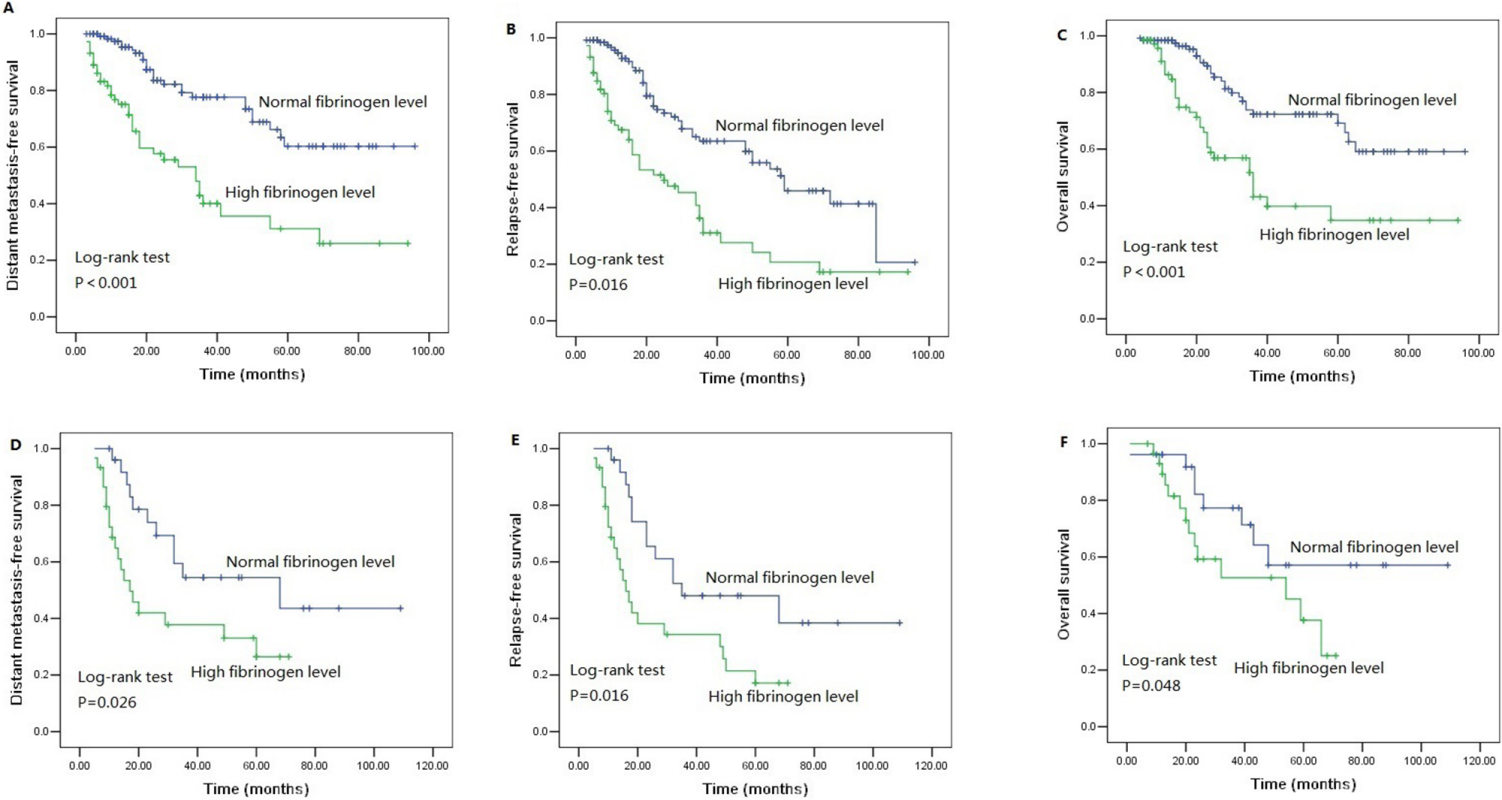
Survival curves of **A.** DMFS **B.** RFS **C.** OS subdivided by plasma fibrinogen level in ESCC patients without adjuvant postoperative radiotherapy (*n* = 199), **D.** DMFS **E.** RFS **F.** OS subdivided by plasma fibrinogen level in ESCC patients with adjuvant postoperative radiotherapy (*n* = 56).

**Figure 5:**
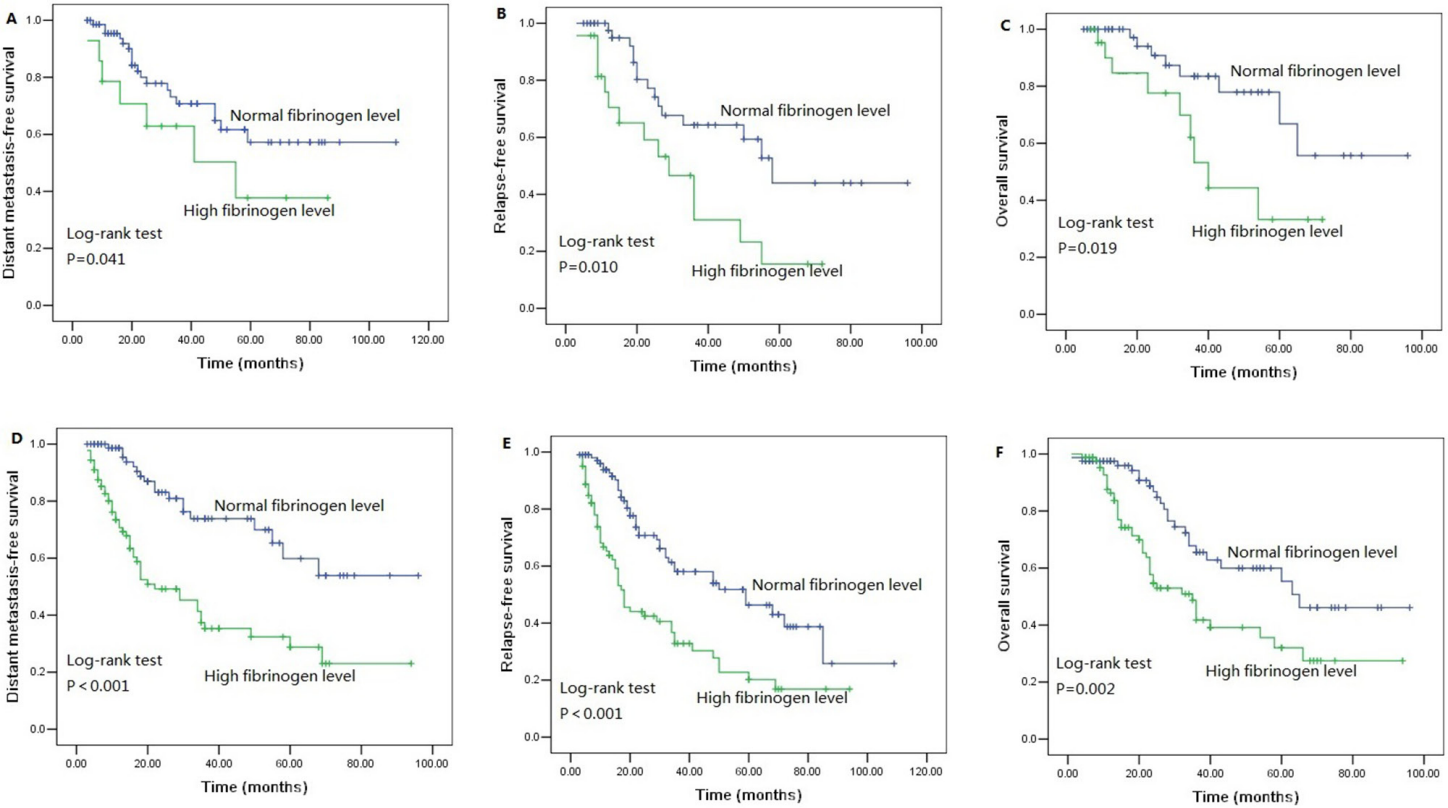
Survival curves of **A.** DMFS **B.** RFS **C.** OS subdivided by plasma fibrinogen level in T_1–2_ stage ESCC patients (*n* = 85), **D.** DMFS **E.** RFS **F.** OS subdivided by plasma fibrinogen level in T_3–4_ stage ESCC patients (*n* = 170).

**Figure 6:**
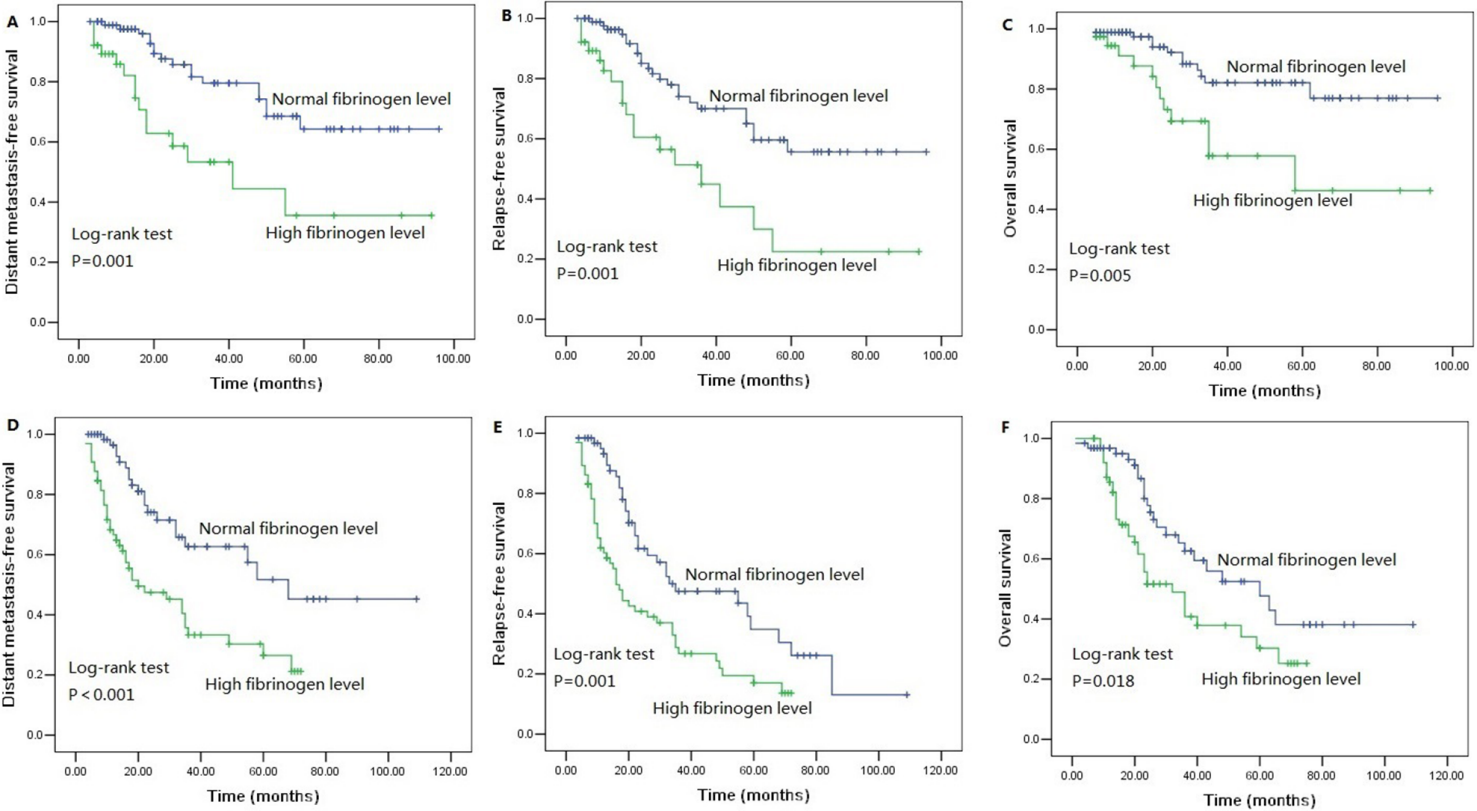
Survival curves of **A.** DMFS **B.** RFS **C.** OS subdivided by plasma fibrinogen level in ESCC patients without lymph node metastasis (*n* = 127), **D.** DMFS **E.** RFS **F.** OS subdivided by plasma fibrinogen level in ESCC patients with lymph node metastasis (*n* = 128).

We performed univariate analysis for plasma fibrinogen level and other nine clinicopathological variables to find out the useful prognostic factors. The results were shown in Table 2. Univariate analysis for LRFS showed that advanced T stage (*P* = 0.041) and regional lymph node metastasis (*P* = 0.024) were two risk factors for poor LRFS. Tumor length, T stage, N stage and plasma fibrinogen level were four significant prognostic factors for DMFS (Tumor length: *P* = 0.009, T stage: *P* = 0.031, N stage: *P* = 0.001, plasma fibrinogen level: *P* < 0.001), RFS (Tumor length: *P* = 0.017, T stage: *P* = 0.004, N stage: *P* < 0.001, plasma fibrinogen level: *P* < 0.001) and OS (Tumor length: *P* < 0.001, T stage: *P* < 0.001, N stage: *P* < 0.001, plasma fibrinogen level: *P* < 0.001). Additionally, tumor cell differentiation was found to have a statistically significant correlation with OS (*P* = 0.033). The patients in the cohort with hyperfibrinogenemia exhibited decreased DMFS (*P* < 0.001; Figure 2A), RFS (*P* < 0.001; Figure 2B) and OS (*P* < 0.001; Figure 2C) compared with the patients who had normal-level plasma fibrinogen. However, plasma fibrinogen level was not significant prognostic factor associated with LRFS (*P* = 0.995; Figure 2D). Furthermore, subgroup analysis according to different clinicopathological variables indicated that DMFS, RFS and OS were shorter in patients with hyperfibrinogenemia (*P* < 0.05, Figure 3, 4, 5, 6).

**Table 2:** Univariate analysis for predictions associated with LRFS, DMFS, RFS and OS in 255 ESCC patients

Variables	LRFS			DMFS			RFS			OS		
	HR	95% CI	*P*	HR	95% CI	*P*	HR	95% CI	*P*	HR	95% CI	*P*
Gender (male/female)	1.02	0.31–3.37	0.971	1.92	0.78–4.72	0.157	1.81	0.84–3.88	0.129	1.64	0.66–4.06	0.283
Age (≥65 Y/<65 Y)	1.36	0.47–3.93	0.567	1.02	0.53–1.97	0.958	1.04	0.58–1.85	0.895	1.19	0.61–2.32	0.605
Smoking (ever/never)	0.79	0.32–1.93	0.606	1.77	0.89–3.53	0.103	1.46	0.84–2.55	0.184	1.50	0.75–3.00	0.253
Alcohol (ever/never)	0.73	0.35–1.52	0.397	1.39	0.86–2.27	0.182	1.31	0.86–1.99	0.205	1.51	0.89–2.59	0.129
Differentiation (poorly/well and moderately)	0.99	0.38–2.57	0.979	1.47	0.90–2.41	0.128	1.38	0.89–2.15	0.151	1.75	1.05–2.93	**0.033**
Tumor location (lower/upper and middle)	1.17	0.58–2.35	0.660	1.16	0.77–1.75	0.476	1.20	0.84–1.72	0.315	1.20	0.78–1.86	0.411
Tumor length (>5 cm/≤5 cm)	0.93	0.35–2.42	0.874	1.85	1.16–2.93	**0.009**	1.66	1.10–2.51	**0.017**	2.49	1.57–3.94	**<0.001**
T stage (T3–4/T1–2)	2.40	1.04–5.57	**0.041**	1.65	1.05–2.60	**0.031**	1.80	1.20–2.68	**0.004**	2.82	1.61–4.95	**<0.001**
N stage (N+/N−)	2.37	1.12–5.02	**0.024**	2.03	1.32–3.12	**0.001**	2.15	1.47–3.13	**<0.001**	2.71	1.67–4.40	**<0.001**
Plasma fibrinogen levels (High/normal)	1.00	0.48–2.08	0.995	3.03	2.00–4.61	**<0.001**	2.48	1.73–3.55	**<0.001**	2.60	1.67–4.04	**<0.001**

Bold values are statistically significant (*P* < 0.05).

Then the multivariate analysis (Table 3) showed that plasma fibrinogen level and N stage were significantly related to DMFS (plasma fibrinogen level, *P* < 0.001; N stage, *P* = 0.045), RFS (plasma fibrinogen level, *P* < 0.001; N stage, *P* = 0.006) and OS (plasma fibrinogen level, *P* = 0.007; N stage, *P* = 0.025). With respect to DMFS and OS, Patients with hyperfibrinogenemia had a 2.541-fold and 1.728-fold increased relative risk of developing distant metastasis and death compared with patients with normal plasma fibrinogen level.

**Table 3:** Prognostic factors for LRFS, DMFS, RFS and OS by multivariate Cox regression analysis in 255 ESCC patients

	LRFS		DMFS		RFS		OS	
	HR (95% CI)	*P*	HR (95% CI)	*P*	HR (95% CI)	*P*	HR (95% CI)	*P*
Differentiation (poorly/well and moderately)	0.987 (0.380–2.566)	0.979	1.469 (0.895–2.413)	0.128	1.381 (0.889–2.146)	0.151	1.834 (1.095–3.073)	**0.021**
Tumor length (>5 cm/≤5 cm)	0.925 (0.354–2.418)	0.874	1.364 (0.849–2.201)	0.204	1.301 (0.848–1.997)	0.228	1.961 (1.211–3.174)	**0.006**
T stage (T3–4/T1–2)	1.958 (0.819–4.679)	0.131	1.039 (0.636–1.700)	0.878	1.208 (0.785–1.860)	0.390	1.797 (0.989–3.264)	0.054
N stage (N+/N−)	1.995 (0.920–4.330)	0.081	1.592 (1.010–2.509)	**0.045**	1.740 (1.170–2.588)	**0.006**	1.997 (1.203–3.304)	**0.007**
Plasma fibrinogen levels (High/normal)	0.998 (0.479–2.077)	0.995	2.541 (1.615–4.001)	**<0.001**	2.002 (1.360–2.946)	**<0.001**	1.728 (1.070–2.793)	**0.025**

Bold values are statistically significant (*P* < 0.05).

## DISCUSSION

Fibrinogen, converted to insoluble fibrin by activated thrombin, plays a key role in the blood clotting, fibrinolysis, inflammatory response, wound healing, and neoplasia [9]. There have been few previous studies describing the role of fibrinogen during the early onset of ESCC. Qi et al employed proteomic profiling to identify markers that correlated with esophageal malignant transformation. Fibrinogen gamma-A chain precursor was found to have more than five-fold difference in expression between immortal and malignant phenotypes [10]. Therefore, fibrinogen was applicable for early diagnosis in ESCC. However, this study was limited by small sample size. In our study, the proportion of hyperfibrinogenemia was higher in ESCC patients than those in healthy controls (40.4% vs 13.6%). These results showed that plasma fibrinogen level could be served as an important role in the diagnosis of ESCC.

In the setting of ESCC, 34% of patients developed hyperfibrinogenemia before surgery and increased plasma fibrinogen level was associated with pathological T stage and lymph node metastasis [11]. Matsuda et al established a FA score based on preoperative plasma fibrinogen and serum albumin levels in esophageal cancer patients and to investigate the correlation with overall survival. Patients with elevated fibrinogen and decreased albumin levels were allocated a score of 2, those with only one of these abnormalities were allocated a score of 1, and those with neither of these abnormalities were allocated a score of 0. The result showed that preoperative FA score was significantly correlated with postoperative survival in esophageal cancer [12]. Recently, Wang et al [13] investigate the clinical significance of preoperative plasma fibrinogen level and platelet count in esophageal squamous cell carcinoma (ESCC) treated by curative surgery. The incidence of hyperfibrinogenemia was 43.7% (52/119) and hyperfibrinogenemia was found to be positively correlated with disease recurrence. However, preoperative plasma fibrinogen level was not an independent prognostic indicator in multivariate analysis. Our data demonstrated that preoperative plasma fibrinogen level together with N stage was an independent factor related with distant organ metastasis for ESCC. Furthermore, shorter DMFS, RFS and OS were also observed in the patients with higher preoperative plasma fibrinogen level. Patients with pretreatment hyperfibrnogenemia had 2.541 times the risk of distant organ metastasis, 2.002 times the risk of disease progression and 1.728 times the risk of death of those with normal pretreatment fibrinogen level. High pretreatment plasma fibrinogen level was significantly associated with decreased DMFS, RFS and OS probability among patients with regional lymph node metastasis and without regional lymph node metastasis (*P* < 0.05). Although plasma fibrinogen level was significantly higher in T3–4 patients than that in T1–2 patients (*P* < 0.05), high pretreatment plasma fibrinogen level was correlated with poorer DMFS, RFS and OS both among T3–4 patients and T1–2 patients (*P* < 0.05). Our finding revealed that pretreatment plasma fibrinogen level was significantly associated with tumor length, tumor location and patient's gender. These results demonstrated that fibrinogen might be important for the acquisition of migration and invasion capabilities of ESCC tumor cells, which subsequently resulted in distant organ metastasis and poorer outcome in ESCC patients. The large number of cases, multivariate analyses, and sufficient survival data make our results more reliable, reproducible, and representative of the studied population.

Currently, there is no general consensus on the role of adjuvant therapy in patients with ESCC. Postoperative treatment modalities depend on tumor invasion, status of regional lymph node metastasis and patients' preference. Regional lymph node is an important factor in determining adjuvant therapy after complete resection. Patients with positive regional lymph node metastasis could benefit on disease free survival and overall survival from adjuvant chemotherapy. Postoperative adjuvant radiotherapy conferred to survival benefit to the ESCC patients with three or more positive regional lymph node metastasis [14, 15]. According to the guideline of National Comprehensive Cancer Network (NCCN), adjuvant chemoradiotherapy is recommended for the ESCC patients with positive regional lymph node metastasis. A Japan Clinical Oncology Group trial (JCOG 9204 trial) showed that postoperative adjuvant chemotherapy can prolong disease-free survival [16]. In a recently meta-analysis, patients with stage III-IV disease or lymph node metastasis could benefit from postoperative adjuvant chemotherapy [17]. In contrast, our study indicated that if patients with longer tumor length, advanced T stage, lymph node metastasis and hyperfibrinogenemia, they could be at high risk of distant organ metastasis and might be recommended adjuvant postoperative chemotherapy. Although, a prospective study is required to determine this strategy, it might help oncologist to select the appropriate treatment for individual patients in advance to prevent metastasis.

In conclusion, we found that elevated plasma fibrinogen levels were associated with significantly elevated risk of ESCC. Plasma fibrinogen level, together with tumor length, T stage and N stage is related with postoperative distant organ metastasis. Preoperative hyperfibrinogenemia is a negative prognostic factor for survival of patients with ESCC. However, it still merits further experimental and clinical investigations to confirm its prognostic significance.

## MATERIALS AND METHODS

A total of 255 patients with ESCC were diagnosed and treated in Zhejiang cancer hospital, Hangzhou, China, between October 2006 and December 2009. All patients were newly confirmed to have ESCC and had not received treatment previously. Patients with the following characteristics were excluded from our study: patients with previous or coexisting cancer other than ESCC; those with concomitant disease suspected of influencing plasma fibrinogen level, such as severe hypertension, liver disease and blood coagulation disorders; and patients who had taken aspirin or other acetylsalicylic acids within 1 month before the treatment; Patients had not undergone complete resection with negative margins. Patients who had undergone exploratory thoracotomy without resection were also excluded from our study. The extent of the disease was determined by TNM staging according to the seventh edition of the International Union Against Cancer (UICC) TNM classification. Our study was approved by the institutional review board of the hospital. All patients provided informed consent before surgery. Population controls were selected from a pool of cancer-free subjects in the same region as cases. The control subjects were frequency-matched to the cases on gender, age and smoking status. Detailed information was obtained by trained interviewers using a structured questionnaire. Finally, a total of 273 eligible subjects enrolled in our study.

All patients underwent total or subtotal transthoracic esophagectomy and regional lymphadenectomy with curative intent. Fifty-five (21.6%) were treated with adjuvant platinum-based chemotherapy. Fifty-six patients were treated with adjuvant locoregional radiotherapy. All patients received standardized follow-up at a 3-month interval for the first 2 years after operation, a 6-month interval in the third year and yearly thereafter. Evaluation comprised a physical examination, upper gastrointestinal endoscopy, complete blood count, chest and abdomen computed tomography.

Locoregional relapse-free survival (LRFS) was defined as the duration from the date of surgery to the date when local or regional relapse was diagnosed. Distant metastasis-free survival (DMFS) covered the date of definitive surgery to the date of distant metastasis was diagnosed. Relapse-free survival (RFS) was defined as the time from surgery to any recurrence. Overall survival (OS) was calculated as the time from the date of surgery to death or censoring.

Each patient and cancer-free people provided 3-mL pretreatment blood. Plasma was separated within 30 min after the blood samples were collected. Fibrinogen was measured by the Clauss method using Diagnostica Stago equipment and reagent according to Diagnostica Stago guidelines (Asnieres, France). According to the instructions, plasma fibrinogen concentration of less than 4.0 g/L was considered normal level, and concentration of ≥4.0 g/L was defined as hyperfibrinogenemia (also as high level). The eligible patients were divided into two groups: normal group and the high level group, according to the above cutoff value.

### Statistical analysis

Plasma fibrinogen concentration was analyzed as a continuous variable and a categorical variable after grouping by normal level and hyperfibrinogenemia. The chi-square test was performed to evaluate the association between the clinicopathological variables and plasma fibrinogen level.

The chi-square test was used to assess the frequencies in select demographic variables, patients' gender and smoking status, between the cases and controls. Patients' age was compared using unpaired student's *t*-test. The association between fibrinogen status and ESCC risk was evaluated by computing the odds ratios (ORs) and 95% confidence intervals (CIs) from multivariate unconditional logistic regression analysis with adjustment for gender, sex and smoking status.

We also used the chi-square test to compare the locoregional relapse, distant metastasis, overall relapse, and mortality rates between the patients with normal plasma fibrinogen level and patients with hyperfibrinogenemia. LRFS, DMFS RFS and OS were analyzed by Kaplan-Meier analysis with log-rank test. Multivariate Cox proportional hazards regression model with forward stepwise approach was constructed to identify independent prognostic factors. All statistical calculations were performed with SPSS 13.0 for Windows (Chicago, IL). *P* < 0.05 were considered statistical significance.

## References

[1] Napier KJ, Scheerer M, Misra S (2014). Esophageal cancer: A Review of epidemiology, pathogenesis, staging workup and treatment modalities. World journal of gastrointestinal oncology.

[2] Wijnhoven BP, Tran KT, Esterman A, Watson DI, Tilanus HW (2007). An evaluation of prognostic factors and tumor staging of resected carcinoma of the esophagus. Annals of surgery.

[3] Quint LE, Hepburn LM, Francis IR, Whyte RI, Orringer MB (1995). Incidence and distribution of distant metastases from newly diagnosed esophageal carcinoma. Cancer.

[4] Diakowska D (2013). Cytokines association with clinical and pathological changes in esophageal squamous cell carcinoma. Disease markers.

[5] Chen M, Huang J, Zhu Z, Zhang J, Li K (2013). Systematic review and meta-analysis of tumor biomarkers in predicting prognosis in esophageal cancer. BMC cancer.

[6] Dipasco PJ, Misra S, Koniaris LG, Moffat FL (2011). Thrombophilic state in cancer, part I: biology, incidence, and risk factors. Journal of surgical oncology.

[7] Diao D, Zhu K, Wang Z, Cheng Y, Li K, Pei L, Dang C (2013). Prognostic value of the D-dimer test in oesophageal cancer during the perioperative period. Journal of surgical oncology.

[8] Shimada H, Oohira G, Okazumi S, Matsubara H, Nabeya Y, Hayashi H, Takeda A, Gunji Y, Ochiai T (2004). Thrombocytosis associated with poor prognosis in patients with esophageal carcinoma. Journal of the American College of Surgeons.

[9] Mosesson MW (2005). Fibrinogen and fibrin structure and functions. Journal of thrombosis and haemostasis : JTH.

[10] Qi YJ, He QY, Ma YF, Du YW, Liu GC, Li YJ, Tsao GS, Ngai SM, Chiu JF (2008). Proteomic identification of malignant transformation-related proteins in esophageal squamous cell carcinoma. Journal of cellular biochemistry.

[11] Takeuchi H, Ikeuchi S, Kitagawa Y, Shimada A, Oishi T, Isobe Y, Kubochi K, Kitajima M, Matsumoto S (2007). Pretreatment plasma fibrinogen level correlates with tumor progression and metastasis in patients with squamous cell carcinoma of the esophagus. Journal of gastroenterology and hepatology.

[12] Matsuda S, Takeuchi H, Kawakubo H, Fukuda K, Nakamura R, Takahashi T, Wada N, Saikawa Y, Omori T, Kitagawa Y (2015). Cumulative prognostic scores based on plasma fibrinogen and serum albumin levels in esophageal cancer patients treated with transthoracic esophagectomy: comparison with the Glasgow prognostic score. Annals of surgical oncology.

[13] Wang J, Liu H, Shao N, Tan B, Song Q, Jia Y, Cheng Y (2015). The clinical significance of preoperative plasma fibrinogen level and platelet count in resectable esophageal squamous cell carcinoma. World journal of surgical oncology.

[14] Xiao ZF, Yang ZY, Miao YJ, Wang LH, Yin WB, Gu XZ, Zhang DC, Sun KL, Chen GY, He J (2005). Influence of number of metastatic lymph nodes on survival of curative resected thoracic esophageal cancer patients and value of radiotherapy: report of 549 cases. International journal of radiation oncology, biology, physics.

[15] Xiao ZF, Yang ZY, Liang J, Miao YJ, Wang M, Yin WB, Gu XZ, Zhang DC, Zhang RG, Wang LJ (2003). Value of radiotherapy after radical surgery for esophageal carcinoma: a report of 495 patients. The Annals of thoracic surgery.

[16] Ando N, Iizuka T, Ide H, Ishida K, Shinoda M, Nishimaki T, Takiyama W, Watanabe H, Isono K, Aoyama N, Makuuchi H, Tanaka O, Yamana H, Ikeuchi S, Kabuto T, Nagai K (2003). Surgery plus chemotherapy compared with surgery alone for localized squamous cell carcinoma of the thoracic esophagus: a Japan Clinical Oncology Group Study—JCOG9204. Journal of clinical oncology : official journal of the American Society of Clinical Oncology.

[17] Zhang SS, Yang H, Xie X, Luo KJ, Wen J, Bella AE, Hu Y, Yang F, Fu JH (2014). Adjuvant chemotherapy versus surgery alone for esophageal squamous cell carcinoma: a meta-analysis of randomized controlled trials and nonrandomized studies. Diseases of the esophagus : official journal of the International Society for Diseases of the Esophagus / ISDE.

